# Food anaphylaxis: data from registry of Center for Severe Allergic Reactions of Piemonte region (Italy)

**DOI:** 10.1186/2045-7022-1-S1-O46

**Published:** 2011-08-12

**Authors:** Alberto Raie, Sabrina Mietta, Enrico Heffler, Gianni Cadario, Maurizio Galimberti, Giovanni Rolla

**Affiliations:** 1 University of Torino, Ospedale Mauriziano Umberto I, Allergology and Clinical Immunology, Torino, Italy; 2 AOU SG Battista of Torino, Allergology and Clinical Immunology, Center for Severe Allergic Reactions of Piemonte Region, Torino, Italy; 3 Ospedale Maggiore e della Carità, Allergology and Clinical Immunology, Novara, Italy

## Background

Key challenges to the study of anaphylaxis are a lack of widely accepted standard working definitions, inadequate reporting of events, failure to agree on a severity threshold for classification as an anaphylactic reaction.

## Aim

To estimate the prevalence of food-allergy anaphylaxis based on the database of the Piemonte Region (Italy) Reference Center for Severe Allergic Reactions.The registry monitors a population of 4,400,000 inhabitants and collects data mandatory for prescribing self-injectable epinephrine reimbursed by Regional Health System.

## Methods

Anaphylaxis cases were diagnosed according to NIAID/FAAN criteria, and assigned to one of three levels of decreasing probability using a clinical checklist based on recommendations of the Brighton Collaboration.

## Results

Among the 1315 reported cases of anaphylaxis, 541 could be classified as food anaphylaxis, with level 1 (38%), level 2 (59%), and level 3 (3%) of probability. 212 patients were children (< 18yrs, age 7.4±5.4 yrs, M/F=2.0), and 329 were adults (age 35.5±12.9 yrs, M/F= 0.5). The main implicated food allergens were nuts (31%), egg (16%), milk (15%), fish (8%) and sesame (7%) in children and nuts (26%), vegetables (14%), crustaceans (12%), fresh fruit (10%), fish (7%), legumes (6%), seeds (6%) and flours (5%) in adults.

Food-dependent exercise-induced anaphylaxis was reported in 28 patients. Skin and respiratory symptoms were reported respectively in 95% and 81% patients, with no differences between children and adults, while gastrointestinal symptoms were more frequent in children (43 vs 29%, p=0.001) and cardiovascular involvement was more frequent in adults (36 vs 16%, p<0.0001).

## Conclusion

Food is an important cause of anaphylaxis, particularly in children (78.8% of all cases) with predominance in boys; this gender preference reverses in adulthood. Egg and milk were specific causes of anaphylaxis in children, while plant-derived foods and crustaceans were more specific in adults. Nuts and fish were triggering allergens in all ages.

Checklists and glossary of terms are crucial to harmonize the report of anaphylaxis cases to a surveillance system or epidemiological study.

